# The Parasitoid *Eretmocerus hayati* Is Compatible with Barrier Cropping to Decrease Whitefly (*Bemisia tabaci* MED) Densities on Cotton in China

**DOI:** 10.3390/insects11010057

**Published:** 2020-01-17

**Authors:** Xiaoming Zhang, Marco Ferrante, Fanghao Wan, Nianwan Yang, Gábor L. Lövei

**Affiliations:** 1 State Key Laboratory for Biology of Plant Diseases and Insect Pests, Institute of Plant Protection, Chinese Academy of Agricultural Sciences, Beijing 100193, China; zxmalex@ynau.edu.cn (X.Z.); wanfanghao@caas.cn (F.W.); 2 State Key Laboratory for Conservation and Utilization of Bio-Resources in Yunnan, College of Plant Protection, Yunnan Agricultural University, Kunming 650201, China; 3 Mitrani Department of Desert Ecology, Ben Gurion University of the Negev, Sede Boqer Campus, Midreshet Ben Gurion IL-8499000, Israel; marco.ferrante@live.it; 4 CE3C–Centre for Ecology, Evolution and Environmental Changes, Azorean Biodiversity Group, Faculty of Agricultural and Environmental Sciences, University of the Azores, PT-9700-042 Angra do Heroísmo, Portugal; 5 Department of Agroecology, Aarhus University, Flakkebjerg Research Centre, DK-4200 Slagelse, Denmark

**Keywords:** biological control, cantaloupe, sunflower, maize, habitat manipulation, non-chemical pest control

## Abstract

The whitefly, *Bemisia tabaci* (Gennadius) cryptic species Mediterranean (MED), is a destructive insect pest worldwide. In order to contribute to controlling *B. tabaci* by non-chemical methods, we examined the possibility of using a combination of trap/barrier crops and a parasitoid natural enemy in cotton. We performed field experiments using cantaloupe (*Cucumis melo*) and sunflower (*Helianthus annuus*) as trap crops and maize (*Zea mays*) as a barrier crop combined with periodic releases of the parasitoid *Eretmocerus hayati* in Hebei Province, Northern China. All treatments significantly reduced immature whitefly densities. Parasitism rate was significantly higher in cotton plots intercropped with sunflower and with perimeter-planted cantaloupe. Adult whitefly density was negatively related to parasitoid abundance and was significantly lower in cotton plots intercropped with maize than in the control plots. Intercropping was more effective than perimeter-planting at reducing *B. tabaci* densities and increasing yield. Parasitoid dispersal was not hampered by barrier crops, indicating that the two methods of control are compatible. These results contribute to the development of integrated pest management methods against this important pest.

## 1. Introduction

Pest control in modern agriculture has been traditionally reliant on the (initially indiscriminate) use of synthetic pesticides. The emerging problems of pesticide residues, resistance in pests [1], and the damage to natural enemies and various other beneficial arthropods and ecological processes triggered the development of alternative methods of pest control, conceptualized as integrated pest management (IPM) [2]. IPM strategies have been implemented in several countries worldwide and are widely gaining acceptance. For example, IPM is now a mandatory method of plant protection in the European Community [3], but much remains to be further developed and articulated.

IPM uses various methods, among which intercropping and the use of biological control are prominent elements with lower environmental costs than other alternatives [4,5]. Intercropping is a non-chemical pest control method that relies on modifying pest and natural enemy behavior as well as distribution and density by planting two or more crops on the same field in various spatial arrangements [6,7,8]. Pests can also be manipulated by attracting or deterring them [9] or by hampering their dispersal by the use of border plants to protect the crop [10,11]. These are especially promising when dealing with pests that have a history of fast development of resistance to multiple pesticides.

The whitefly complex *Bemisia tabaci* (Gennadius) is a “species flock” widely present in tropical and subtropical areas world-wide [12,13,14]. They cause serious economic damage by direct feeding [15,16,17] but also as vectors of plant viruses [13,18]. The group is very prone to developing resistance to several pesticides [19,20]. This makes chemical protection problematic and thus generated interest in various non-chemical methods. Several tall-growing non-host plants, primarily grasses (Gramineae), have been successfully used to reduce whitefly colonization and virus transmission, including sorghum, *Sorghum bicolor* (L.) Moench [21], pearl millet, *Pennisetum typhoides* (Burm. f.) Stapf & Hubbard [22,23], and maize, *Zea mays* L. [24]. However, the method does not always work; densities of *B. tabaci* and virus incidence can be higher on cassava when intercropped with maize than in monoculture [25]. Trap and barrier cropping experiments in China indicate that intercropping is more effective than perimeter planting to reduce whitefly densities in cotton, and maize is more effective in this role than sunflower or cantaloupe [26]. Nonetheless, whitefly densities can remain above the economic damage threshold, thus the possibility of combining this with other methods of pest control is worth exploring.

Numerous natural enemies have been introduced to various parts of the world to suppress *B. tabaci* [27,28,29,30,31]. In several cases, the released natural enemies successfully control the target pest [32,33]. The parasitoid *Eretmocerus hayati* Zolnerowich and Rose (Hymenoptera: Aphelinidae) is perhaps the most widely used species to manage outbreaks of whiteflies [32]. A host suitability study under laboratory conditions indicates that *E. hayati* has the highest control potential of *B. tabaci* among currently reported parasitoids in China [34]. As a solitary primary parasitoid, *E. hayati* shows high preference for *B. tabaci* on various host plants, including tomato, eggplant, poinsettia, and cotton [32,35,36,37]. Although the release of a parasitoid could suppress whiteflies, this has not always been observed [34,38,39]. In such situations, combination of various methods may offer a solution. However, the successful combined application of natural enemies with intercropping can be constrained by interference between biological control agents and barrier planting methods [40,41]. Here, we explored the possibility of combining barrier and trap cropping with periodic release of this parasitoid to examine the compatibility of these two methods of control.

Specifically, we tested the following predictions:

(1) Whitefly densities would remain lower under intercropping than perimeter planting;
(2) The effect of parasitoids would be additive to the effect of barrier cropping;
(3) These treatments would cause an increase in cotton yield.

## 2. Materials and Methods

### 2.1. Experimental Design and Plot Management

**Figure 1:**
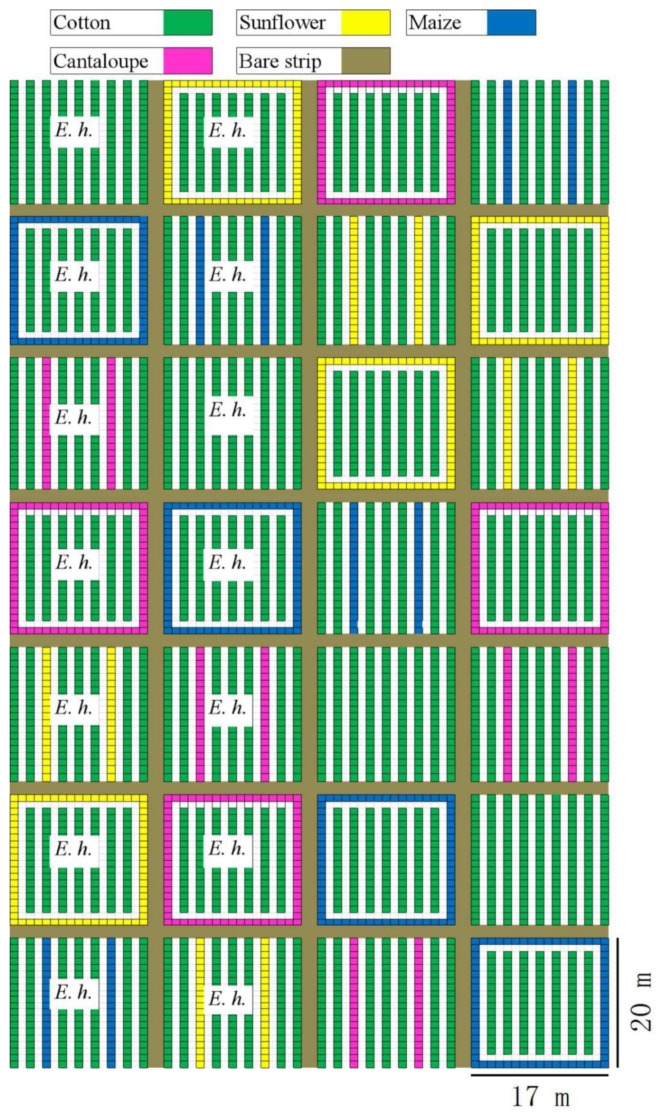
Spatial layout of the field experiment. E. h. indicate that *Eretmocerus hayati* was released in this plot. Plots (17 × 20 m) consisted of nine rows of planting beds, each 20 m long and 1 m wide, separated by 1 m from each other. Each bed was planted with two rows of cotton plants, and each row was planted with 60 plants 33 cm apart. Plots were separated from each other by a 2 m wide bare strip.

The experiments were conducted in 2012 at the Langfang Experimental Station of the Chinese Academy of Agricultural Sciences (CAAS) in Hebei Province (39°30′42″ N, 116°36′07″ E), Northern China. Plot arrangement was the same as in Zhang et al. (2020). Cotton (cv. Zhongmian 6) was planted on 11 May 2012, while cantaloupe (cv. Elizabeth Hybrid F1), maize (cv. Huaiyan 10), and sunflower (cv. CH 609) were planted on 31 May 2012. Due to a catastrophic rain in the area on 21 July 2012, almost all cantaloupe plants died and had to be replanted using seedlings on 7 August 2012 (Figure 1).

Two planting patterns were compared, intercropping and perimeter planting, as detailed in Zhang et al. (2020). The resulting treatments included three intercrop (I) patterns (maize, MI; cantaloupe, CI; and sunflower, SI) and three perimeter (P) plantings (maize, MP; cantaloupe, CP; and sunflower, SP). In the control plot, only cotton was planted. Each treatment had four replicates, and the plots were randomly allocated to treatments.

### 2.2. Parasitoid Rearing

We used the aphelinid *E. hayati*, a solitary parasitoid ovipositing externally under the host nymphs at their 1st–3rd stages [34]. Upon eclosion, the first instar parasitoid larva penetrates the host, feeds, and pupates internally. As the parasitoid larva grows, parasitized nymphs become shiny and yellow, and at a more advanced stage, the eyes of the adult parasitoid wasp are clearly seen through the host skin.

All the host-plants and insects were kept under laboratory conditions at 26 ± 2 °C, 65 ± 5% relative humidity (RH), under a 14 h:10 h L:D regime at Langfang Experimental Station. The laboratory colony of *B. tabaci* cryptic species Mediterranean (MED) was collected from greenhouses at the Institute of Vegetables and Flowers, CAAS, Beijing, China and was maintained under glasshouse conditions without exposure to insecticides for 3 years. The species was kept on tomato plants (*Solanum lycopersicum* cv. He-Zuo 918) obtained from the Shanghai Tomato Research Institute. When tomato seedlings were 10–15 cm tall with 6–8 leaves, they were individually transplanted to flower pots. Then, 6–8 of these pots were put into a *B. tabaci* rearing cage made of 100 mesh nylon gauze (1.0 m long × 0.8 m wide × 1 m tall), and 150 newly emerged adults (sex ratio 1:1) per tomato plant were released into the cage (900–1200 adults) to lay eggs. After 24 h, all the adults were removed. After the emerging *B. tabaci* reached the 2nd nymphal stage (in 12–14 days), we introduced 5 newly emerged, mated female *E. hayati* per tomato plant. The development of parasitism in whiteflies was checked every 3 days. When parasitoid pupae appeared, we transferred all parasitoid rearing cages to another room at 16 ± 1 °C and 75 ± 5% RH, 6 h:18 h L:D regime to delay the development of both host and parasitoid. Cages/pots were kept at this temperature for 15–20 days in preparation for release.

### 2.3. Parasitoid Release

Twenty-four hours before parasitoid release, the potted plants with parasitized whiteflies were transferred to another room with 26 ± 2 °C, 65 ± 5% RH, and a 14 h:10 h L:D regime to prepare the parasitoids for release. At this stage, the numbers were adjusted; a predetermined number of parasitized nymphs were kept on the potted plants, and the rest were removed by hand. After this, the potted plants were placed in the middle of the selected experimental plots. For each treatment, parasitoids were released in two randomly chosen plots, and the other two were kept as release-free controls. The *E. hayati* release time was chosen to coincide with the peak time of whitefly.

Densities were based on our census in 2011 [26] under calm, sunny periods. Numbers released were determined by the amount of parasitoids available at the time. The first release was on 18 July 2012, when six potted tomato plants were placed in each selected cotton plot with 400 parasitized whitefly nymphs on each (total 2400 *E. hayati* pupae/plot). On 30 July 2012, three potted tomato plants with 100 parasitized *B. tabaci* on each were placed out (300 parasitoids/plot), and on 11 August, four potted plants with 150 parasitized *B. tabaci* (600 parasitoids/plot) were released. This way, a potential total of 3300 parasitoids were released in each plot over 3 weeks. Adult parasitoids started to hatch 5–7 days after the plants were placed outdoors.

### 2.4. Sampling

#### 2.4.1. *B*. *tabaci* Population

Field sampling started on 28 June 2012 and was repeated every 10 days until the end of the cotton growing season (8 October 2012). The survey design was identical to Zhang et al. (2020). In each plot, a total of 150 cotton leaves were censused. Adult whiteflies were counted in situ; afterwards, the selected leaves were cut, individually placed in plastic bags, and brought to the laboratory to count nymphs under a dissecting microscope (Olympus, SZ51, 20× magnification). Following this, the area of each leaf was recorded using a transparent mm paper placed over the leaf, from which standardized density data (number of individuals per 100 cm^2^ leaf surfaces) were calculated [42].

The identity of the whiteflies in the study area was checked as in Zhang et al. (2020), and all individuals tested belonged to *B. tabaci* MED.

#### 2.4.2. Parasitoid Census

Censuses were conducted every 10 days using the same leaves as for the whitefly population census on cotton. Adult parasitoids were counted on the spot. After this, the selected leaves were taken back to the laboratory for examination under a dissecting microscope (2× magnification). Whitefly nymphs with displaced myelomas (symbiotic housing organs) were considered parasitized. The nymphs were subsequently maintained with the host leaf petiole soaking in nutrient solution for an additional 5–7 days in the laboratory until parasitism could be ascertained by the yellow coloration of the *E. hayati* pupal stage [43]. The first record of parasitoids on cotton leaves was about 3 weeks after the first release, on 8 August (in CP and SI), but densities were very low (0.036 and 0.181 individuals 100 cm^−2^, respectively). Parasitoids started to be common in all plots from 18 August onwards.

#### 2.4.3. Cotton Yield

Cotton yield was determined on a five-point sampling method [44]. On each plot, 2015 plants were randomly selected, and we counted the number of fruiting branches per plant and the number of cotton bolls per branch on 13 October. The mean mass of a cotton boll was measured on 1520 ad hoc selected bolls, and from this, the mass of cotton bolls per plant was estimated. The mature cotton bolls were harvested on three occasions—13 and 22 October and 20 November—then taken back to the laboratory, briefly dried at room temperature for 24 h, and weighed.

### 2.5. Data Analysis

**Figure 2:**
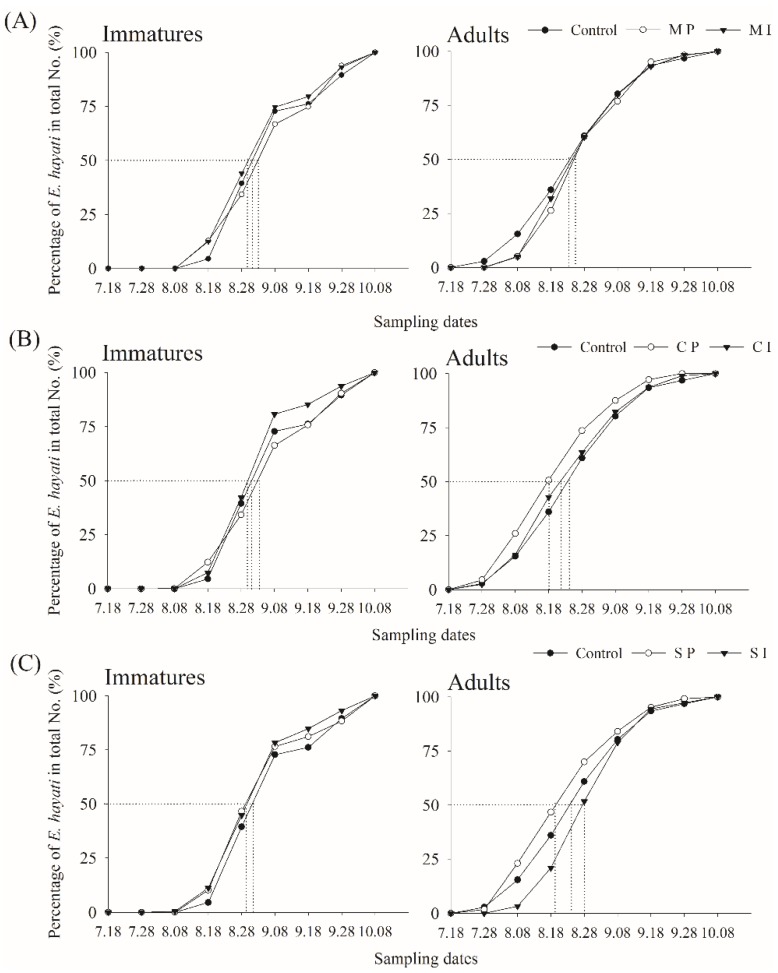
Cumulative seasonal activity curves of *Eretmocerus hayati* in cotton with inter- or perimeter-cropped maize (**A**), cantaloupe (**B**), and sunflower (**C**) at Langfang, Hebei Province, northern China, in 2012.

All statistical analyses were performed with the R program, version 3.3.3 [45], using the packages *glmmTMB* [46] and *lsmeans* [47]. The whitefly periods of activity (early, main, and late) were determined following the method by Fazekas et al. (1997) [48], as in Zhang et al. (2020) (Figure 2). In order to unequivocally discern the effectiveness of barrier/trap crops with and without parasitoid release against adult and immature whiteflies, we first analyzed the effects of barrier/trap crops only in plots without parasitoid release (Section 2.5.1) and later in plots with and without parasitoid release (Section 2.5.2).

#### 2.5.1. Barrier/Trap Crop Effect

Average densities of adult and immature whiteflies on cotton leaves were analyzed in two separate generalized linear mixed models (GLMMs) with a Poisson and a negative binomial distribution, respectively. In the initial model, rounded average densities of whiteflies per plot were used as response, treatment was included as fixed factor, and sampling date and activity period were random factors. These models suffered minor underdispersion (dispersion parameter = 0.65 and 0.86 for adult and immature whitefly densities, respectively). To avoid stronger underdispersion, the final model with adult whitefly densities as response only included treatment and sampling date. Data exploration was carried out according to Zuur et al. (2010) [49] and model validation by investigating Pearson’s residuals [50]. LSMeans Tukey’s HSD post-hoc test was used to identify significant differences (e.g., [51]). 

#### 2.5.2. Barrier/Trap Crop and Parasitoid Release Effect

Average densities of adult and immature whiteflies on cotton leaves were analyzed in two separate GLMMs with a Poisson distribution. The initial model was formulated as for the previous analysis, but it also included parasitoid abundance as a fixed factor. Backward model selection was done by comparing Akaike Information Criterion (AIC) values [52]. The model with adult whitefly densities as response suffered strong underdispersion (dispersion parameter = 0.30), which was impossible to correct. An additional GLMM with Gaussian distribution, parasitism rate (i.e., the proportion of parasitized immature whitefly per sample) as response, treatment as fixed factor, and sampling date and activity period as random factors was tested. Data exploration, model validation, and post-hoc evaluation were done as for the previous analysis.

#### 2.5.3. Cotton Yield

We used ANOVA to test mean yield differences between treatments and the Tukey’s post hoc test to reveal significant effects. 

## 3. Results

### 3.1. Adult and Immature Whitefly and Parasitoid Densities

The average adult density during the entire season was 1.44 individuals 100 cm^−2^ leaf surface (SD = 1.12, *n* = 308). Adult densities were 1.15 ind. 100 cm^−2^ (SD = 1.05, *n* = 112) during the early period, peaked at 2.33 ind. 100 cm^−2^ (SD = 1.11, *n* = 84) during the main, and were reduced to 1.07 ind. 100 cm^−2^ (SD = 0.78, *n* = 112) during the late season. The average immature density during the entire season was 6.8 individuals 100 cm^−2^ (SD = 5.96, *n* = 308). Immature densities were 4.21 ind. 100 cm^−2^ (SD = 4.2, *n* = 112) during the early season, increasing to 13.33 ind. 100 cm^−2^ (SD = 6.19, *n* = 84) during the main season before decreasing to 4.50 ind. 100 cm^−2^ (SD = 2.73, *n* = 112) during the late season. Parasitoid density during the entire season was 0.12 ind. 100 cm^−2^ (SD = 0.12, *n* = 224), showing a similar seasonal trend: 0.02 ind. 100 cm^−2^ (SD = 0.04, *n* = 28) during the early, 0.19 ind. 100 cm^−2^ (SD = 0.13, *n* = 84) during the main, and 0.08 ind. 100 cm^−2^ (SD = 0.09, *n* = 112) during the late season. Parasitoid seasonality did not differ much between the treatments (Figure 2).

### 3.2. Barrier/Trap Crop Effect 

**Figure 3:**
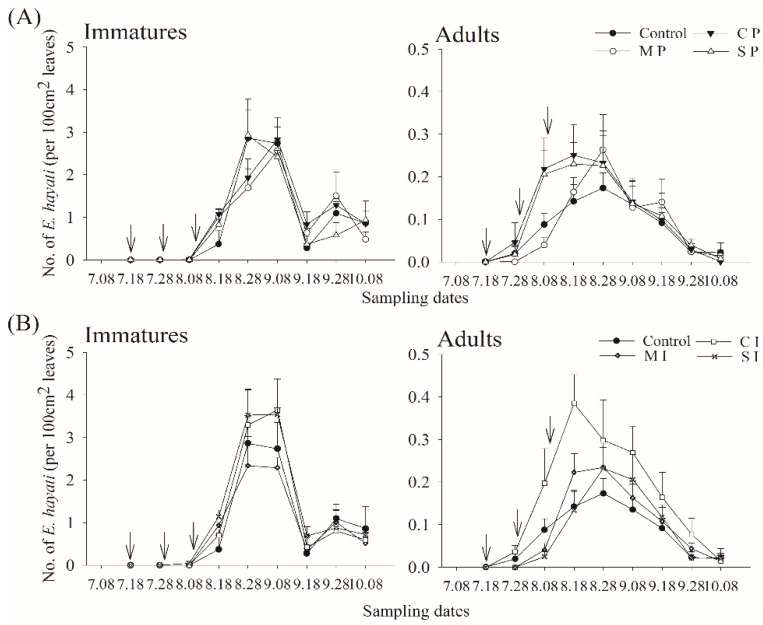
Seasonal dynamics of *Eretmocerus hayati* (mean + SE) on cotton with perimeter- (**A**) or intercropped (**B**) maize, cantaloupe, and sunflower at Langfang, Hebei Province, northern China, in 2012. In upper of the figures ‘*↓*’ means *E. hayati* had released in this date in the fields.

Adult whitefly densities were significantly (LSD Tukey post hoc test, t = 3.317, d.f. = 146, *p* = 0.024) lower in plots intercropped with maize (mean = 1.18 ind. 100 cm^−2^, SD = 0.85, *n* = 22) than in the control (mean = 2.59 ind. 100 cm^−2^, SD = 1.82, *n* = 22). Other treatments did not have a significant effect on the density of adults, although all of them had densities lower than the control. Maize, either intercropped (mean = 6.09 ind. 100 cm^−2^, SD = 5.12, *n* = 22) or planted at the perimeter (mean = 5.50 ind. 100 cm^−2^, SD = 5.44, *n* = 22), and intercropped sunflower (mean = 5.95 ind. 100 cm^−2^, SD = 4.50, *n* = 22) significantly reduced immature whitefly densities (LSD Tukey post hoc test, t = 3.189−3.309, d.f. = 144, *p* = 0.0333, *p* = 0.0024 and *p* = 0.0236, respectively) compared to the control (mean = 8.91 ind. 100 cm^−2^, SD = 6.49, *n* = 22) (Figure 3A,B).

### 3.3. Barrier/Trap Crop and Parasitoid Release Effect

Plots intercropped with maize (mean = 1.27 ind. 100 cm^−2^, SD = 0.70, *n* = 32) or sunflower (mean = 1.36 ind. 100 cm^−2^, SD = 0.81, *n* = 32) had significantly fewer (LSD Tukey post hoc test, t = 3.596 and t = 3.348, d.f. = 214, *p* = 0.008 and *p* = 0.019) adults than the control (mean = 2.58 ind. 100 cm^−2^, SD = 1.50, *n* = 32) (Figure 3B). The density of adult whiteflies was negatively affected by parasitoid abundance (GLMM, z = −2.479, *p* = 0.013). Immature whitefly densities were not affected by parasitoid abundance. However, parasitism rate was significantly higher (LSD Tukey’s post-hoc test, t = 3.263, d.f. = 157, *p* = 0.028 and t = 2.858, d.f. = 157, *p* = 0.097, respectively) in plots intercropped with sunflower (mean = 22.5, SD = 12.5, *n* = 24) and perimeter-planted cantaloupe (mean = 21.3, SD = 11.4, *n* = 24) than in the control (mean = 13.6, SD = 12.8, *n* = 23) (Figure 3A,B).

### 3.4. Yield

The average cotton yield in the control plots (mean = 109.22 g plant^−1^, SD = 4.10, *n* = 4) was significantly lower (Tukey’s post hoc test, t = 4.405 and t = 3.815, d.f. = 21, *p* = 0.004 and *p* = 0.015) than in plots intercropped with cantaloupe (mean = 134.08 g plant^−1^, SD = 11.25, *n* = 4) or maize (mean = 130.75 g plant^−1^, SD = 8.11, *n* = 4). The average yield in plots intercropped with cantaloupe was also significantly higher (Tukey’s post hoc test, z = 3.382, d.f. = 21, *p* = 0.038) than in plots with perimeter-planted sunflower (mean = 114.99 g plant^−1^, SD = 6.35, *n* = 4).

## 4. Discussion

Intercropping was more efficient than perimeter planting in reducing whitefly densities, confirming the findings by Zhang et al. (2020) and our first prediction. In our experiments, parasitoids provided an additional top-down control effect on whitefly densities, indicating that these two methods were compatible in most cases in our study system and supporting our second prediction. In some instances, there were no differences from the control; results varied according to barrier plant or intercropping system, and densities of adults versus immature were occasionally also different. This showed that the parasitoids and the whitefly densities were impacted by the planting microenvironment [9] and the general intensity of pesticide use in the landscape [1]. In many cases, intercropping can increase habitat diversity and increase the ability of natural enemies to control target pests. For example, planting buckwheat can enhance parasitism in broccoli [53], and floral understories in a New Zealand apple orchard enhanced parasitism on leafrollers (Lepidoptera: Tortricidae) [54]. 

However, intercropping does not always work in synergy with natural enemies. In open-field and mesocosm experiments, intercropping collards with parsley limited the effectiveness of natural enemies of aphids [55]. These, however, were non-flying natural enemy species, and their dispersal may have been hampered by the increased habitat complexity [55]. Flying natural enemies may not react to increased above-ground habitat diversity in a similar way. Intercropping squash with buckwheat (*Fagopyurm esculentum*) in Florida, USA resulted in neither improved yield nor lower pest levels [56]. Canola (*Brassica napus* L.)-wheat (*Triticum aestivum* L.) intercrops do not appear to favor parasitism of *Delia radicum* by either *Aleochara bilineata* Gyllenhal or *A. verna* Say (Coleoptera: Staphylinidae) [57]. The dispersal ability of the natural enemy is likely to be an important factor to explain why these two pest control strategies are only sometimes successful together. *Eretmocerus hayati* disperses mainly at random and with the help of the wind [58]. Maize is a tall plant, and cotton plots with intercropped and perimeter-planted maize may constrain the dispersal ability of this parasitoid. The reduced parasitism rates we found in cotton plots intercropped with sunflower and with perimeter-planted cantaloupe, which are smaller plants, suggest that parasitoid release may be effective in controlling whiteflies densities in these settings.

Cantaloupe, by attracting whiteflies, can reduce whitefly densities in cotton, but its effectiveness is influenced by the planting pattern [26,59]. An interesting difference was the impact of cantaloupe on whitefly densities, which performed well in these experiments but was not similarly effective earlier [26]. The difference was possibly due to the serendipitous weather event, the torrential rain in July 2012 that killed off the earlier-planted cantaloupe. Consequently, these replanted plants were younger than during the previous experiments. This underlines the importance of the timing for such plants; the barrier plants probably act as physical barriers, and thus the taller they are, the more effective they can be. The vegetative state of these plants clearly influences their effect on the target pest, and the attraction may be linked to plant stimuli produced at a younger developmental stage. Further studies could fine-tune the optimal planting time to maximize the intended attractant effect. 

Increasing crop diversity can provide more favorable micro-environment, food, alternative host, or prey resources for natural enemies [60,61]. In particular, nectar-producing plants can increase the diversity and the fitness of natural enemies, thereby increasing their number and their effect on pest populations [62,63]. The attractant approach relies mainly on the plant volatiles, and other plants constitute a physical barrier effect to reduce pest densities, but they could also act through increased farmland biodiversity, ultimately supporting several ecosystem services simply by increasing spatial heterogeneity at the plot/farm scale [64].

The fact that the relative effectiveness of the various barrier and trap crops in reducing whitefly densities were not the same on plots with and without parasitoid release may indicate that parasitoids were not a simple addition to the management system. Possibly, they reacted to the plot environment, including the chemical stimuli from the plants, and modified the final pest densities by their differential reaction. The precise nature of this interaction also needs further study.

From the point of practical benefits, we again proved that using these methods will result in higher per-plant yield, supporting our third prediction.

## 5. Conclusions

Our results indicated that the two non-chemical protection measures, barrier planting and parasitoid release, were compatible in reducing whitefly densities in cotton fields. Parasitism rate is probably influenced by the numbers released in augmentative biological control as well as the level of pesticide use in the landscape, as we found that parasites also invaded plots where they were not released. Nonetheless, the employed pest management methods brought clear yield benefits and are promising in the development of non-chemical methods in cotton production.
